# Predicting noncontact injuries of professional football players using machine learning

**DOI:** 10.1371/journal.pone.0315481

**Published:** 2025-01-02

**Authors:** Diogo Nuno Freitas, Sheikh Shanawaz Mostafa, Romualdo Caldeira, Francisco Santos, Eduardo Fermé, Élvio R. Gouveia, Fernando Morgado-Dias

**Affiliations:** 1 Interactive Technologies Institute (ITI/LARSyS), Funchal, Portugal; 2 Faculty of Exact Sciences and Engineering, University of Madeira, Funchal, Portugal; 3 NOVA Laboratory for Computer Science and Informatics, Caparica, Portugal; 4 Department of Physical Education and Sport, University of Madeira, Funchal, Portugal; Deakin University, AUSTRALIA

## Abstract

Noncontact injuries are prevalent among professional football players. Yet, most research on this topic is retrospective, focusing solely on statistical correlations between Global Positioning System (GPS) metrics and injury occurrence, overlooking the multifactorial nature of injuries. This study introduces an automated injury identification and prediction approach using machine learning, leveraging GPS data and player-specific parameters. A sample of 34 male professional players from a Portuguese first-division team was analyzed, combining GPS data from Catapult receivers with descriptive variables for machine learning models—Support Vector Machines (SVMs), Feedforward Neural Networks (FNNs), and Adaptive Boosting (AdaBoost)—to predict injuries. These models, particularly the SVMs with cost-sensitive learning, showed high accuracy in detecting injury events, achieving a sensitivity of 71.43%, specificity of 74.19%, and overall accuracy of 74.22%. Key predictive factors included the player’s position, session type, player load, velocity and acceleration. The developed models are notable for their balanced sensitivity and specificity, efficiency without extensive manual data collection, and capability to predict injuries for short time frames. These advancements will aid coaching staff in identifying high-risk players, optimizing team performance, and reducing rehabilitation costs.

## Introduction

Professional football players are confronted with high physical and mental demand levels. This condition inevitably leads to a substantial likelihood of (noncontact) injury events [1]. In this study, a noncontact injury is defined in alignment with the Union of European Football Associations (UEFA) guidelines for epidemiological research. Specifically, it refers to an acute physical pain experienced by a player during a training session or match, which occurs without any physical contact with other players. Such an injury may or may not necessitate medical intervention but results in the player being unable to participate in the subsequent training session or match [2, 3].

Noncontact injuries (henceforth, injuries) are known to significantly impact the player’s physical performance and psychological aspects, with repercussions for the entire team [4] and club [5, 6]. These injuries account for more than a third of all injuries that require players to stop, and more than a quarter of all injuries reported in a season [7, 8]. Nevertheless, the scientific evidence of the last two decades around the modifiable risk factors associated with preventing and reducing the risk of injury in professional football players is increasing [9].

One way of assessing the risk of injury is by employing screening battery processes [6, 10, 11] or training load monitoring [12, 13]. In training load monitoring, Global Positioning Systems (GPS) receivers occupy a prominent place since they can be used to measure distances covered by players, and in conjunction with other sensors, quantify the number of accelerations and decelerations, as well as provide estimations of measurements of the global and metabolic external training load.

Previous studies have investigated the relationship between training planning based on GPS metrics and the risk of injury [14–17]. The majority of these studies are retrospective in nature and primarily focus on identifying significant statistical correlations between individual GPS-derived metrics and the incidence of injuries. Nevertheless, the causal relationship of injuries is complex and influenced by multiple factors. This means that while certain metrics tracked by GPS devices might show a correlation with the occurrence of injuries under specific conditions, there are also other important metrics that might not seem significant on their own. However, when these metrics are analyzed together with additional data, they could become important indicators of injury risk [18, 19]. In response to that multifaceted task, the literature suggests using machine learning techniques as an imminent solution for injury prediction [20]. Nevertheless, certain studies concentrate on predicting specific types of injuries. Ayala et al. [19], for example, explored the potential of these techniques by using pre-season evaluation data, including personal, psychological, and neuromuscular measurements, to predict hamstring strain injuries. Similarly, Ruiz-Pérez et al. [21] leveraged machine learning to predict lower extremity soft tissue injuries in elite futsal players. In both scenarios, the predictive models generate an estimation of the likelihood that each player will sustain an injury over the course of the entire season.

In a different view, several studies have investigated the capabilities of machine learning methods in predicting injuries throughout a football season. A noteworthy example is the work of Rossi [22], which not only proposed a machine learning-based injury predictor but also introduced an interpretable framework for understanding the underlying causes of injuries. On the other hand, several studies [23–25] emphasize the importance of integrating diverse data sources, such as manually collected questionnaire responses and GPS tracking data, to improve the accuracy of machine learning predictions. In particular, Vallance et al. [24] successfully developed an injury predictor capable of making short-term (1-week) and medium-term (1-month) predictions. The authors utilized GPS tracking data alongside data from well-being questionnaires as inputs for various machine learning models.

The existing literature provides only a preliminary understanding of the factors that influence the risk of injury [26], and the statistical prediction models used are still basic (e.g., cut-off values [26]) or not accurate enough [11, 27] (i.e., not able to correctly identify both injury and noninjury events). Machine learning strategies, on the other hand, are associated with a high incidence of false positives. This could result in coaches erroneously sidelining players who are incorrectly flagged as having a high risk of injury or misallocation of medical resources. Additionally, existing methods focus exclusively on a singular injury type, do not provide precise temporal information about injury risk, and necessitate continuous manual data collection to enhance the accuracy of predictive models.

The primary goal of this study is to address these challenges. More specifically, this research is aimed to develop an automated system that uses machine learning techniques to predict injury risks. Unlike previous machine learning models designed for injury prediction, the proposed approach calculates the likelihood of injury for each player daily over the course of a football season. To achieve this, data from GPS devices are utilized in conjunction with variables related to the players and the match sessions. This study employed the Maximum Relevance–Minimum Redundancy (mRMR) [28] in combination with a wrapper method for feature selection, which helps in identifying the most relevant and nonredundant features for predicting injury events.

It is important to note here that the methodology employed does not utilize data from questionnaires or any other continuous manual data collection.

More specifically, the objectives of this paper are to:

- identify the most relevant metrics with minimal redundancy for predicting injury events. Improved understanding and the elimination of overlapping features (i.e., redundant) can enable coaches to concentrate solely on the most critical features to better prevent injuries while improving player performance.
- develop predictive models based on Adaptive Boosting (AdaBoost), Feedforward Neural Network (FNN), and Support-Vector Machine (SVM) classifiers, considering a trade-off between accurately detecting true injury events and decreasing the likelihood of incorrectly identifying noninjury events as injuries (i.e., false positives).
- propose a strategy for incorporating the effects of sudden changes in player load into the predictive models. This approach aims to provide a more accurate assessment of injury risk by accounting for fluctuations in physical demands on athletes.

## Materials and methods

**Fig 1:**
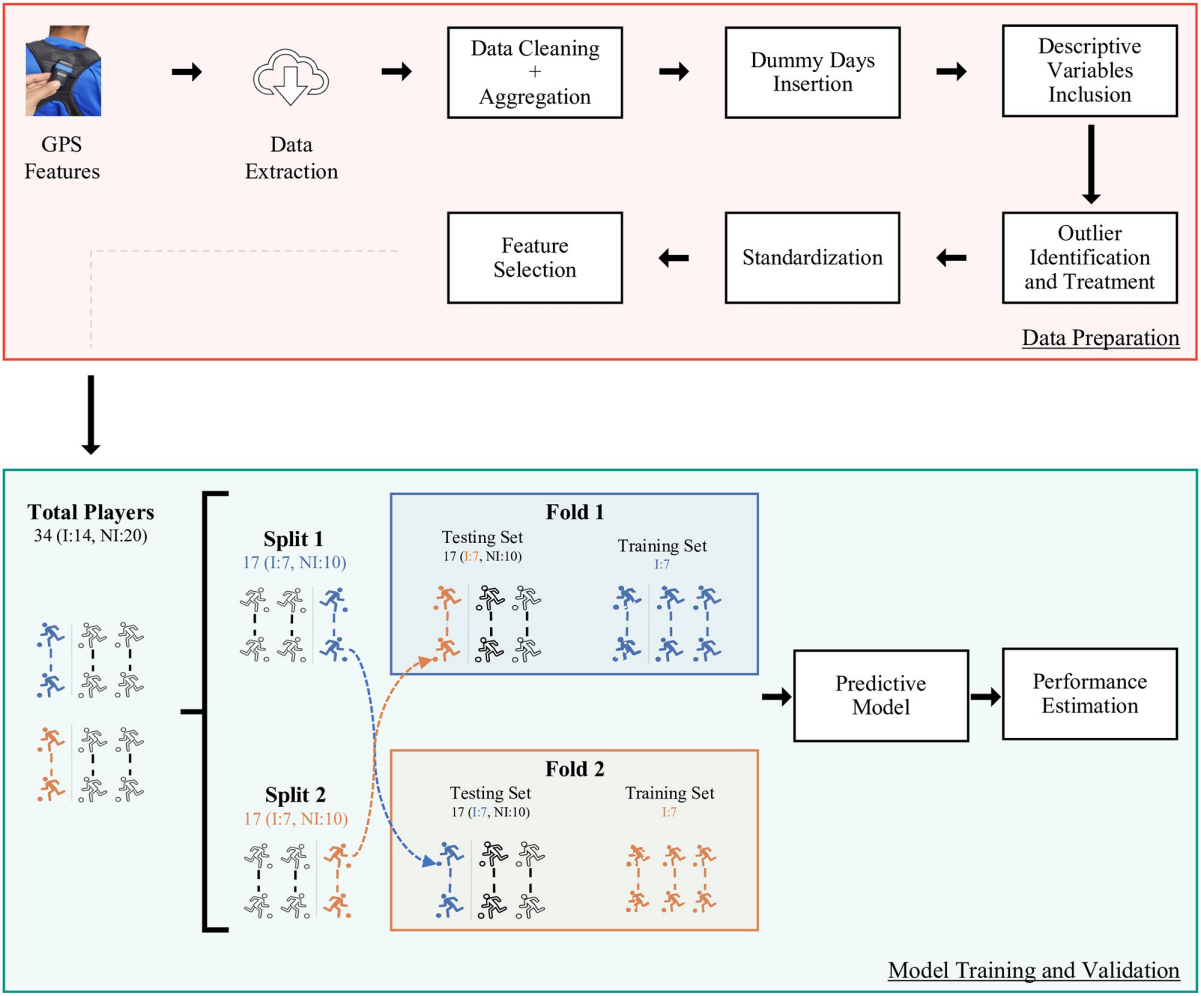
Machine learning pipeline used in the study for injury detection. The process begins with data collection, followed by data preparation. Subsequent stages involve the development and validation of predictive models to ensure accuracy and reliability in injury detection.

Fig 1 depicts the machine learning pipeline followed in the current study for injury detection. All the steps of this pipeline will be explored in the following sections.

### Design and data collection

A longitudinal study was conducted among a convenience sample of 34 male professional football players from a Portuguese first-division team. The team’s physical trainers collected data over 36 weeks in the 2020–2021 season, covering 217 training sessions and 38 official games. The data collection period was from September 7, 2020, to May 19, 2021.

Participants had a mean age of (26.27 ± 3.28) years, a mass of (77.54 ± 7.63) kg (collected with the InBody 770), and a height of (180.65 ± 6.60) cm measured with a stadiometer (SECA 213). These professional players were distributed among five positions. That is, 11 (32.35%) players were defenders, 9 (26.47%) attacking midfielders, 7 (20.59%) forwards, 4 (11.76%) defensive midfielders, and 3 (8.82%) midfielders. Besides being a goalkeeper, no other exclusion criterion was applied to recruit the players for this study.

The data was collected in the context of the football club’s professional contract with each player. These contracts between the players and the club included clauses for gathering data related to their performance, thus ensuring that consent for this activity was formally obtained. As already mentioned, the responsibility for collecting these data was assigned to the club’s physical trainers, meaning the researchers were not involved in the data collection phase. Nevertheless, the authors submitted a request for an ethical assessment to obtain access to this data. This request was approved by the Faculty of Human Kinetics ethics committee at the University of Lisbon (statement no. 34/2021). The committee’s approval verified that the study complied with national and international ethical standards, as outlined in the Convention on Human Rights and Biomedicine (Oviedo Convention) and the Declaration of Helsinki. Furthermore, all participants provided written informed consent, ensuring they were willingly participating and fully aware of the study’s goals and methods. After receiving ethical approval and informed consent, the researchers accessed the data on June 15, 2021, which marked the start of the data analysis phase.

The club’s medical staff reported 18 traumatic and overload injuries throughout the 2020–2021 season. Of these 18 injuries, 10 (55.56%) injury events were due to match circumstances, and 8(44.44%) were during training sessions. Moreover, most of the injuries were located in the muscles and tendons (11 injuries, 61.11%), the remaining being in the ligaments (6 injuries, 33.33%) or contusions (1 injury, 5.56%).

Players used GPS receivers from Catapult (GPSports EVO) placed in a skin-tight vest in the thoracic region between the scapulae, capturing the players’ position data with a sampling frequency of 10 Hz. Such devices were already in use by the team when the study started. Despite this prior use, they have been shown to be valid, reliable, and accurate for measuring acceleration [29, 30] and the mechanical work applied to the player [12]. Moreover, since these devices are equipped with accelerometers, magnetometers, and other sensors, it was possible to collect raw data, which was then processed by the Catapult system to derive a total of 1379 parameters for this study. The average Horizontal Dilution of Precision (HDOP) recorded was 1.12, which serves as a metric to assess the geometric accuracy of the GPS satellite-based positioning. HDOP values span from 1 to 2, with values closer to 1 reflecting higher positional accuracy.

### Data preparation

All the players’ metrics data per exercise and session were collected by the GPS receivers and extracted using the Catapult’s GPSports Cloud. The information was then grouped (using the proper aggregation function, such as maximum and average) into a single record according to the player’s unique identifier and session date.

Moreover, metrics from GPS that contained GPS information (e.g., maximum satellite count and HDOP), as well as invalid or missing data were removed. The latter includes, e.g., metrics with invalid data due to sensors’ absence (such as heart rate and players’ weight).

The unique identifier for each player was also removed from the dataset to maintain anonymity. This step also ensures that the prediction models were trained without access to player-specific information, allowing the models to remain player-independent.

Those processes resulted in a set of 424 GPS metrics that were retained for further data preprocessing steps.

#### Dummy days insertion

In order for the model to capture sudden changes in the players’ load, time information called “dummy days” was added after grouping the sessions by player and date. A dummy day is attributed to a specific athlete and is denoted by a series of zeroes across all measured variables, thereby emulating a hiatus in the athlete’s training regimen, which may arise from intervals of rest or recovery.

In other words, a dummy day is a record that indicates a specific day with no physical activity records from a given player. This information will help the models capture sudden changes in the player’s load time series, a potential factor that increases the injury risk [31]. Also, this strategy enables the predictive models to learn short-term dependencies between past and current period information.

Furthermore, the inclusion of dummy days ensures a continuous time scale in the dataset for all players, allowing the model to consistently analyze the temporal sequence of training and rest periods.

#### Descriptive variables inclusion

Besides all the parameters collected by the GPS receivers, it was possible to identify other descriptive variables that could increase the probability of a given player having an injury. Namely, (a) the players’ position and corridor [32]; (b) the players’ age (in months) at the time of the session or match [11]; (c) the day of the week; (d) the type of session (i.e., training or match session); (e) the results of the games (win, draw or loss); (f) the game location (own stadium or opponent’s stadium); (g) the competition of the match session (in this case, Liga NOS [first division] or Taça de Portugal [national championship]); (h) the number of exercises that the players’ did in a session and (i) the duration of the session.

It is also important to note that before feeding the information (GPS parameters and descriptive variables) to the models, categorical parameters were converted to numerical values utilizing the one-hot encoding method [33].

The study utilized parameters obtained from GPS receivers as well as other descriptive variables as features. Additionally, the variable “injury” was incorporated into the dataset as a binary target variable. Nevertheless, not every feature was retained for the final model; feature selection using the mRMR [28] method was conducted to exclude variables that were either irrelevant or redundant.

#### Outlier identification and treatment

Outliers were identified based on the upper and lower bounds calculated for each GPS parameter values **x**_**i**_ taking into account the data from injured players.

The upper ${x_{i}}^{\text{U}}$ and lower ${x_{i}}^{\text{L}}$ bounds for the *i*-th parameter were computed as

$$\begin{array}{c} {x_{i}}^{\text{L}}={\tilde{x}}_{i}-(\lfloor \frac{{\text{min}}_{i}}{\sigma_{i}}\rfloor +1)\times \sigma_{i}, \end{array}$$ (1)

and

$$\begin{array}{c} {x_{i}}^{\text{U}}={\tilde{x}}_{i}+(\lfloor \frac{{\text{max}}_{i}}{\sigma_{i}}\rfloor +1)\times \sigma_{i} \end{array}$$ (2)

where ${\tilde{x}}_{i}$ denotes the median, *σ*_*i*_ the standard deviation, and min_*i*_ and max_*i*_ the minimum and maximum value of the *i*-th parameter, respectively. The lower and upper bounds were derived from the Standard Deviation Method for identifying outliers [33]. In contrast to the original method, which uses a predetermined number of standard deviations, the approach followed here calculates the number of standard deviations based on the maximum and minimum values of each parameter, ensuring that at least one standard deviation is always maintained.

As a result, the *j*-th data point of the *i*-th parameter was calculated as:

$$\begin{array}{c} x_{i,j}=\{\begin{array}{cc} {x_{i}}^{\text{L}}, & \text{if}\;x_{i,j}<{x_{i}}^{\text{L}}\\ {x_{i}}^{\text{U}}, & \text{if}\;x_{i,j}>{x_{i}}^{\text{U}}\\ x_{i,j}, & \text{otherwise}. \end{array} \end{array}$$ (3)

It is also noteworthy that ${x_{i}}^{\text{U}}$ and ${x_{i}}^{\text{L}}$ were calculated based on data from injured players only. This is because injury events can be caused by abnormal parameter values (compared to noninjury records), and these values should not be considered outliers. The outlier treatment was, however, applied to all records, including noninjured records.

The decision to replace outlier values with the calculated upper or lower bounds was made to maintain the data distribution’s integrity and minimize the distortion of the features’ players’ natural range. In other words, this method maintains the important differences between the data points of a player, which is essential for the machine learning models to correctly identify patterns related to the risk of injury.

#### Standardization

Parameters were separately standardized such that the overall parameters’ values have a mean of zero and a standard deviation of one, that is

$$\begin{array}{c} x_{i}=\frac{x_{i}-{\bar{x}}_{i}}{\sigma_{i}}, \end{array}$$ (4)

where ${\bar{x}}_{i}$ denotes the arithmetic mean.

This process is essential since the parameters are measured in different measurement units. Besides, having all the parameters on the same scale improves the stability of the models during the learning phase [34].

#### Feature selection

In order to reduce the number of input variables of the predictive injury model, and thus reduce the computation time of training the model, a dimensionality reduction was performed by eliminating all features that demonstrated zero variance (i.e., constant variables). In total, 449 features were processed in this step of the machine learning pipeline (424 GPS features and 25 descriptive variables), resulting in 189 features being removed. In other words, 260 features (237 GPS features and 23 descriptive variables) were kept for further investigation.

After removing all features exhibiting zero variance, an additional feature selection analysis was conducted to obtain the most important features for the injury detection model. In this case, the analysis consisted of using the mRMR method [28] to calculate the importance of each feature and rank the features based on their importance. Subsequently, the most important features were identified, and the top *p*-features were selected for inclusion in the injury detection model.

mRMR [28] is a model-agnostic feature selection mechanism that finds an optimal set of features that minimizes the redundancy between the independent variables, and at the same time, maximizes the relevance with respect to the dependent variable (in this case, with the injury). The mRMR algorithm also ranks the features according to their importance and redundancy. Therefore, in this study, the method was used only to sort the GPS and the descriptive parameters, and only the top features were used in the models.

In order to determine the ideal number of features for each injury detection model, and thus perform feature selection, the models were tested using various feature sets derived from the standardized dataset with outliers removed. Each set of features was composed of the first *p* (where *p* = 10, 20, ⋯, 260) most important and less redundant features according to the mRMR method. The combination of this wrapper feature selection mechanism with the mRMR method is a novel approach in the field of automatic injury prediction and is significantly faster than a sequential forward search [35].

S1 Table provides a comprehensive list of the GPS and the descriptive parameters collected in this study. The supplementary table also includes their ranking according to the mRMR method.

### Predictive models

This work used SVMs, FNNs, and AdaBoost classifiers to map the GPS parameters and the descriptive variables to the recorded injury events. In other words, the predictive models were fed the most important GPS parameters (with dummy days, followed by outlier treatment and standardization) and combined with the most important descriptive variables (such as player position and corridor). It is highlighted again that, in order to simplify the data representation, the players’ data per exercise and session were grouped into a single record per day, i.e., each player has one sample per day.

Each data point in the dataset has been assigned a discrete label based on the occurrence of an injury event for a given player and day (i.e., 1 = injured, 0 = noninjured). The predictive models utilize these labels to make accurate injury predictions. Although the injury labels presented to the models are binary, the models’ output is a continuous prediction in the range of (0, 1). Nevertheless, applying a threshold can convert these continuous predictions to a discrete label.

That procedure opens the opportunity to adapt this threshold according to the strategy defined for the team. For instance, decreasing the threshold at the start of the season can make the model more sensitive to potential injury events, leading coaches to reduce training sessions’ intensity in order to have more players free from injury during this period. On the other hand, at the end of the season or in preparation for big games, the football coaches, coaching staff, and sports science staff could opt to increase this threshold, balancing better preparation (with higher training loads) with an increased risk of injury. It is important to note here that to report the results of our approach, the threshold was selected using the Receiver Operating Characteristic (ROC) curve. The ROC curve is a graphical representation that illustrates the trade-off between sensitivity and the False Positive Rate (FPR) at various threshold values. The selected threshold was the point on the ROC curve nearest to the top-left corner. This point corresponds to the highest sensitivity and minimum FPR, and is often referred to as the “elbow” of the ROC curve, signifying the location where the sensitivity and specificity (1—FPR) balance is optimal. It is also important to highlight that thresholds were calculated on the training sets and subsequently applied to the testing sets.

The task of predicting injury events presents an imbalanced machine learning problem since only 0.20% of the observations correspond to injury events within the total dataset. For this reason, each model was trained using both cost-sensitive and traditional learning (i.e., cost-insensitive learning) in a supervised manner. In cost-sensitive learning, misclassification errors have different costs depending on the class, which leads to minimizing the total cost during model training. This enables the models to learn how to classify minority classes correctly (in this study, the injury class) [36]. On the other hand, traditional learning does not explicitly address class imbalance, theoretically making the models less prone to classify the minority class correctly. In the cost-sensitive models, the cost of each class was inversely proportional to class frequencies.

For the case of the SVMs, the calculated class weights were used to adjust the margin proportionally. For AdaBoost, the weights were applied to increase the influence of misclassified instances, ensuring that the model focuses more on injury records. Similarly, for FNNs, the class weights were applied to adjust the weighted squared error loss function, making the model more attentive to injury records during training and enhancing its sensitivity to these instances.

The stratified cross-validation [37] method was used to validate the predictive models. This was necessary due to the class imbalance between injured and noninjured records. Additionally, the scarcity of injury records required an effective validation method, as creating a validation set with the required number of samples was not possible.

The cross-validation strategy splits the data into two subsets (i.e., two-fold), as depicted in Fig 1. Each subset holds a random training dataset and a test data set. In both folds, the training datasets contain only players who were injured at least once during the season (50% of the total players injured in each fold). The subsets are subject-independent, meaning that each player’s data is exclusively in only one of the dataset splits. In other words, the cross-validation strategy was implemented on a player-by-player basis, meaning that the division of data was conducted with respect to individual players rather than individual samples.

As a result, the stratified cross-validation yielded split sets of 1501 samples for the training set and 3090 for the testing set in fold 1, with 11 and 7 injury records (i.e., where injury = 1) included in the training and testing sets, respectively. On the other hand, for fold 2, the training set comprised 1410 records, and the testing set comprised 3082. As expected, the training and testing sets for fold 2 contain, respectively, 7 and 11 injury records.

It is important to highlight that the train and test datasets were created from the complete dataset after executing outlier removal, standardization, feature selection, and feature importance sorting. To help address the class imbalance, however, an undersampling technique was applied to both training sets using the *k*-means clustering algorithm before being fed to the models. Essentially, the *k*-means clustering method undersamples noninjured records (i.e., samples where injury = 0) by replacing them with a cluster centroid calculated on noninjured records. The ratio of noninjured records to injured records was set at 40% and *k* = 8.

All the predictive models were built using Python (v. 3.8) with the TensorFlow [38] (v. 2.3.0) and scikit-learn [39] (v. 0.24.2) libraries, in conjunction with other supplementary libraries, such as pandas [40]. Models were trained in a machine equipped with an Intel Core i7–9700F CPU, running at 3.00 GHz, with 32GB of RAM.

#### Support-vector machine

A SVMs is a supervised learning algorithm that constructs an optimal hyperplane through an optimization strategy. This hyperplane is designed to maximize the margin between the data points of distinct classes; specifically, in the context of this work, it differentiates between noninjured and injured records. SVMs are known for their good generalization capabilities, robustness, and effectiveness, even in high-dimensional spaces. Additionally, they require low computational requirements [41]. Besides that, they are commonly used for injury forecasting [42].

In order to develop an SVM-based injury model, the selected regularization parameter was the squared L2 penalty, and the radial basis function kernel was employed. Also, the classification output of the SVM was transformed into probability by using the Platt scaling [43], making thus possible to use a custom threshold for the final binary classification.

#### Feedforward neural network

A FNNs is a directed graph that processes input data through weighted connections, optimized during training to approximate the desired output. FNNs were chosen for this work mainly due to their ability to learn complex and nonlinear relationships between the inputs (i.e., the multiple predictor variables) and the desired output [44]. Besides that, although FNNs have shown to be successful in various sport science works (see, e.g., [45, 46]), their application to injury prediction is still to be further explored [10, 20]. In fact, to the authors’ best knowledge, only the work developed by Ruddy et al. [44] used FNNs for injury prediction (in this case, injuries from professional Australian footballers).

For this work, FNNs with three layers were used. The number of units on the input layer was tuned by testing the different number of features. The hidden layer consisted of ten units with the hyperbolic tangent activation function. A single neuron unit with the sigmoid function was used for the output layer with the range of (0, 1). A dropout regularization technique was also applied between the hidden and output layers [47]. The dropout probability was set to 60% to prevent overfitting due to the limited data available for feeding the model. Lastly, and similarly to the SVM models, the chosen regularization parameter is the squared L2 penalty.

The FNNs were trained over 20 epochs using the Adam [48] optimization algorithm with a learning rate of 0.001, being the loss function the binary cross-entropy.

#### AdaBoost

AdaBoost is a well-known meta-learning algorithm in the boosting family due to its capabilities. The algorithm combines multiple weak classifiers into a strong one by iteratively adjusting the weights of training samples based on the performance of previous classifiers. AdaBoost has already been successfully applied for injury prediction; however, only two works were reported to the authors’ best knowledge [10, 19]. For this reason, exploring the AdaBoost algorithm in other contexts was considered necessary.

AdaBoost is often used with decision trees (which was also the case for this work), where the learning models are stumps—one-level decision trees [49]. The stumps are added to the ensemble at each iteration to minimize the errors from the previous weak learners [50]. A maximum of 50 stumps were added to the final model.

## Results

Sensitivity is the ability of the model to identify injuries correctly, and specificity is the ability of the model to identify noninjuries correctly. The average Geometric Mean (GMEAN) was calculated between the two folds to find a balance between sensitivity and specificity. This function is defined as

$$\begin{array}{c} \text{GMEAN}={\left(\text{Sensitivity}\times \text{Specificity}\right)}^{\frac{1}{2}}, \end{array}$$ (5)

with higher values representing better models.

The features sorted by the mRMR method were tested for the best combination by sequentially adding features to the models. In order to achieve statistical significance, each combination of model, type of learning, and number of features was tested 500 times, resulting in 78 000 simulations.

The results of the current work are divided into three sections. The first section investigates the effect of each type of learning on the GMEAN value. Then, the best models found among the simulations are presented. Finally, the last section assesses the quality and stability of those models.

### Cost-sensitive learning and traditional learning

**Fig 2:**
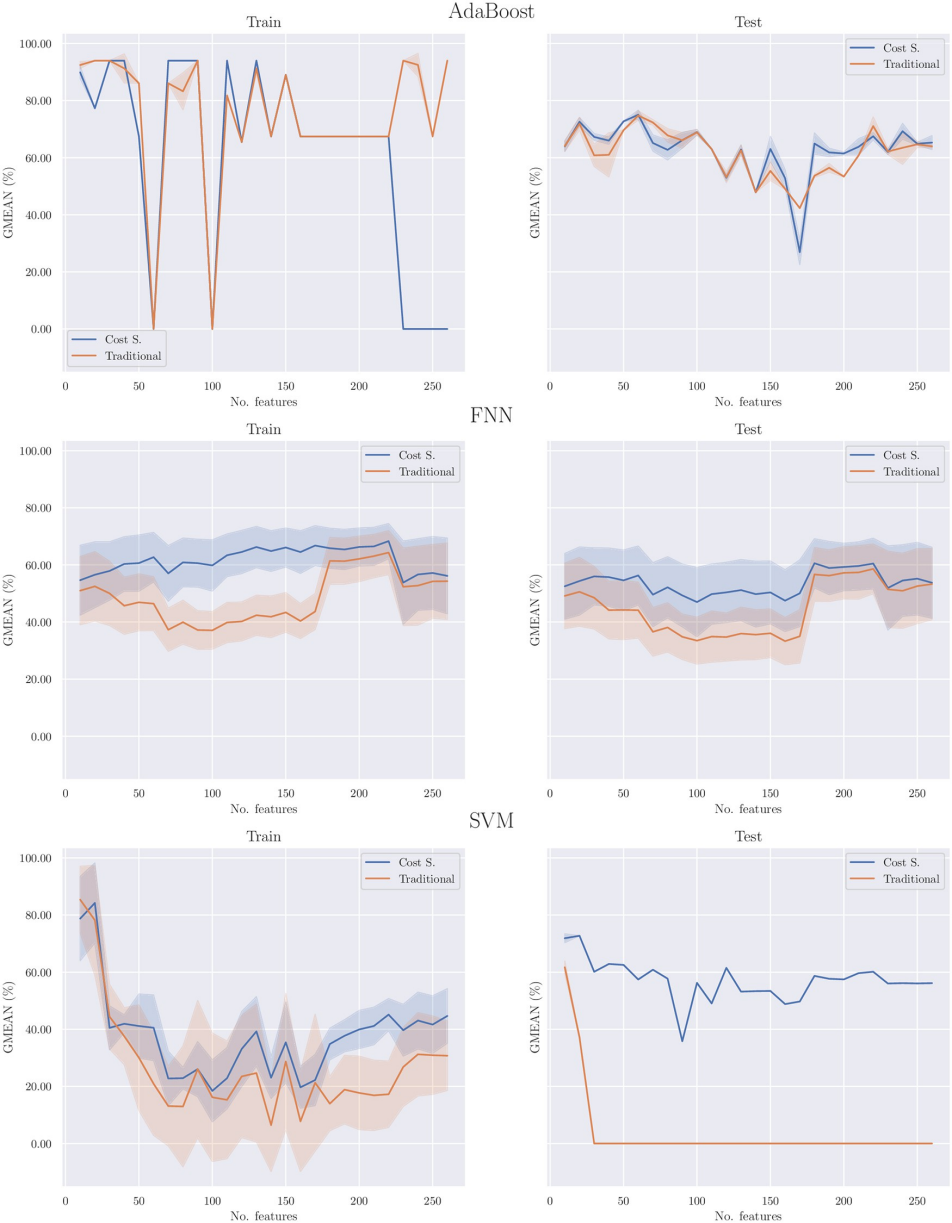
Comparison of cost-sensitive and traditional learning approaches across 500 simulations. This figure depicts the comparison of models’ average GMEAN values from 500 simulations, contrasting cost-sensitive learning with traditional learning methods for both train and test data splits. It also illustrates the impact of adding more features on the GMEAN scores of classifiers. The shaded areas represent the standard deviation, highlighting the variability within each model configuration.

Fig 2 compares, for each model, the cost-sensitive learning and traditional learning approaches regarding the average GMEAN value of the 500 runs for each number of features. It also offers the possibility of studying the number of features each model requires to reach a certain GMEAN level.

From that figure, it is possible to infer that cost-sensitive learning in the AdaBoost models does not represent a substantial advantage in terms of average GMEAN value. Regarding the training splits, if the number of features is lower than 230, both types of learning behave similarly; nevertheless, if a higher number of features are fed to the models, the traditional learning models are, on average, significantly better than the cost-sensitive learning models. In the testing splits, both types of learning follow the same trend of average GMEAN values. Nevertheless, traditional learning is better in some number of features and cost-sensitive in others (especially in the range of 180 to 210 features).

For the FNN and SVM models, however, cost-sensitive learning was always the best methodology to train the models. For these models, the average GMEAN value was consistently superior to traditional learning. This situation can be stated in both training and testing splits.

It is also important to note that for the AdaBoost and SVM models, both types of learning require approximately the same number of features to reach a certain level of average GMEAN value. On the contrary, FNNs trained with a cost-sensitive learning approach needed fewer features when compared to traditional learning. As an illustration, in order to reach 60.00% of the average GMEAN value on the training splits, cost-sensitive learning requires 40 features, while traditional learning requires 180 (127.27% more).

### Best predictive models

The best predictive models were selected in a three-step process. First, the best classifiers were selected by considering the highest value of GMEAN in the training splits for each model and type of learning (independently of the number of features). Then, the same models were tested on data from players that were not used to train the models (i.e., unseen data). The final best models were then selected considering the highest GMEAN in the testing splits. It is highlighted again that, due to data limitations, it was not possible to create a validation set that would further improve the selection of the best models.

Those models are depicted in Table 1, along with their respective evaluation metrics and the best number of features. From this table, it can be concluded that the best two models used the AdaBoost learning method. Regardless of the type of learning, these classifiers obtained in the training splits a mean sensitivity of 88.31%, a specificity of 100.00%, and an accuracy of 96.60%, resulting in a GMEAN of 93.97%.

**Table 1:** Evaluation metrics of the best classifiers found. These classifiers were chosen considering the highest geometric mean value, calculated between sensitivity and specificity on the training splits, for each type of learning and classifier.

Cost-sensitive	AdaBoost		FNN		SVM	
	Yes	No	Yes	No	Yes	No
No. features	90	240	120	220	20	150
**Train**						
Sensitivity (%)	88.31	88.31	83.77	76.62	88.31	88.31
Specificity (%)	100.00	100.00	85.81	86.26	94.44	98.15
Accuracy (%)	96.60	96.60	85.80	86.21	92.65	95.29
AUC	1.00	1.00	0.88	0.87	0.98	0.99
GMEAN (%)	93.97	93.97	84.78	81.30	91.33	93.10
**Test**						
Sensitivity (%)	78.57	86.36	35.06	57.79	71.43	0.00
Specificity (%)	65.02	58.31	81.81	82.19	74.19	100.00
Accuracy (%)	65.08	58.38	81.67	82.13	74.22	99.71
AUC	0.72	0.73	0.46	0.75	0.85	0.73
GMEAN (%)	71.47	70.96	53.56	68.92	72.80	0.00

Followed by the AdaBoost classifiers, SVMs were the second-best models, obtaining a GMEAN ranging from 91.33% to 93.10 in the training splits. Although the AdaBoost and SVMs models presented similar sensitivity values (88.31%), i.e., an equivalent capacity of identifying injuries on truly injury events, the mean specificity metrics dropped 5.56% in cost-sensitive learning and 1.85% in traditional learning.

In its turn, FNNs were the models with the lowest performances, with a GMEAN ranging from 81.30% to 84.78% Consequently, these classifiers also obtained the lowest mean sensitivity and specificity metric values in the training splits (76.62% and 85.81%, respectively).

After choosing the best classifiers in the training datasets, the models that best predicted the injury and noninjury events on the testing splits were the AdaBoost and SVM, both combined with cost-sensitive learning. If, on the one hand, the AdaBoost classifier obtained a GMEAN value of 71.47%, on the other hand, the SVM obtained the more balanced result (balancing sensitivity and specificity), reflected in the highest GMEAN value obtained (72.80%). At the same time, the SVM model only required 20 features (vs. 90 features required by the AdaBoost classifier) to predict more than 70% of injury and noninjury events. Nevertheless, these two models were selected as the best two models of this work since they achieved similar GMEAN values.

It is noteworthy, however, that the AdaBoost classifier combined with traditional learning was the model with the highest sensitivity (86.36%) at the cost of having a low specificity and accuracy. This is a common scenario in imbalanced classification problems. At the other end of the spectrum, the model incorporating SVM with traditional learning could not detect any injury, invalidating its use in real-world scenarios.

Similarly to the training splits, FNN got one of the lowest performances of the set of models and types of learning. Indeed, the ability of the FNN trained using cost-sensitive learning to detect injuries is even inferior to a pure random classifier. The combination of FNN with traditional learning is, moreover, at the limit of its use in a real-world application since it can only detect 57.79% of the actual injury events. Nevertheless, both models presented a high percentage of correct detections of noninjury events (81.81% and 82.19%, respectively).

The effect of cost-sensitive learning on the evaluation metrics compared to traditional learning is visible here. In this work, all models trained with cost-sensitive learning resulted in sensitivities in the training splits equal to or higher than traditional learning. As expected, the models’ accuracies were neglected in the training splits; in the testing splits, it is interesting to note that traditional learning seems more favorable in correctly predicting injury events. However, the more balanced results (i.e., the higher GMEAN values) are usually achieved with cost-sensitive learning coupled with fewer features.

### Quality and stability of the models

**Fig 3:**
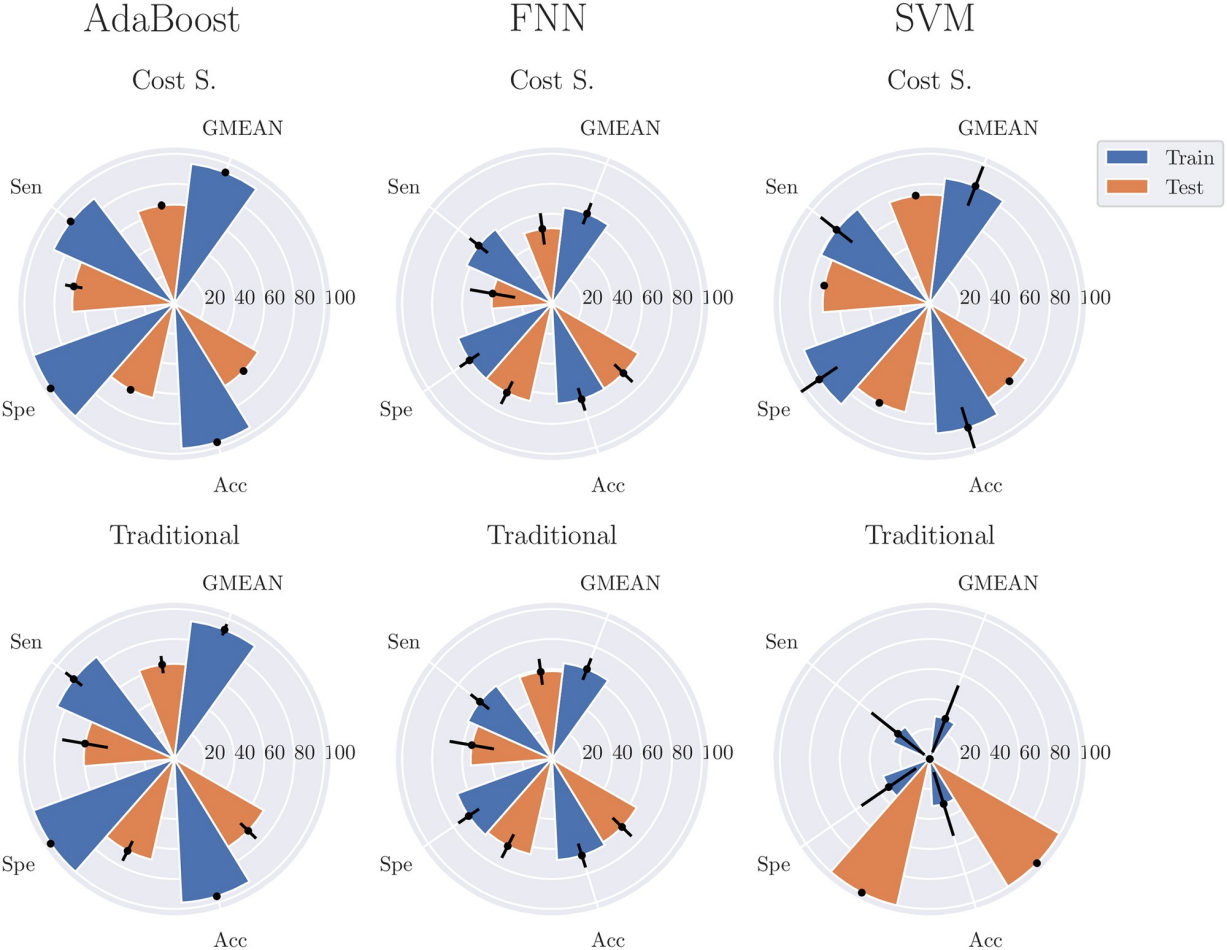
Radar plots of model performance metrics across 500 simulations. The radar plots presented here compare the quality and stability of various models and learning types over 500 simulations, based on the best combinations of model, learning type, and feature count as identified in Table 1. Stability is assessed through standard deviations depicted as lines on top of the bars, while quality is measured in terms of GMEAN values, sensitivity (Sen), specificity (Spe), and accuracy (Acc) for both training and testing data splits.

Fig 3 depicts the radar plots containing the mean and standard deviation information obtained from the 500 runs of the models’ combinations, type of learning, and number of features identified in Table 1. This figure thus enables assessing the quality and stability (based on the standard deviation) of the injury forecasting models in the training and testing phases. At the same time, it also compares cost-sensitive learning with traditional learning, in terms of GMEAN, accuracy, specificity, and sensitivity, for both train and test splits.

AdaBoost models trained with cost-sensitive learning consistently learned to predict 88.31% of the injury events in the training splits. Although the 500 trials exhibit, during training, the same and constant evaluation metric values from Table 1, the same was not true for the testing phase, especially for the sensitivity metric. In testing, these models correctly predicted, on average, 68.09% (SD = 5.82%) of the injury events and 64.33% (SD = 0.51%) of the noninjury events, resulting in an average GMEAN value of 66.11% (SD = 2.72%).

Cost-sensitive learning SVMs models, on the other hand, were shown to predict, on average, 79.43% (SD = 13.43%) and 71.43% (SD = 0.00%) of the injury events in the training and testing phases, respectively. Unlike the previous AdaBoost models, SVMs trained with cost-sensitive learning were more stable in the testing phase than in the training phase in all evaluation metrics. For instance, the specificity obtained in the training splits was 89.36% with a standard deviation of 14.62%; conversely, in the testing phase, a specificity of 74.10% with a standard deviation of 0.03% was reached. It is also important to note the average GMEAN values, which were 84.25% (SD = 14.01%) for training and 72.75% (SD = 0.01%) for testing.

AdaBoost models trained using traditional learning gave more emphasis to specificity and accuracy at the expense of a lower sensitivity value in the testing splits. Still, these models were able to detect correctly, on average, 60.47% (SD = 15.42%) of the injury events and 68.76% (SD = 7.35%) of noninjury events. On the other hand, FNN models were again dubious concerning their applicability in real scenarios, only detecting 40.25% (SD = 15.24%) and 54.32% (SD = 15.00%) of the actual injury events when using cost-sensitive learning and traditional learning, respectively. It is also noteworthy that, from the 500 trials, no SVM model trained using traditional learning was able to detect true injury events.

Overall, models trained with cost-sensitive learning showed to predict, as expected due to this learning’s sensitivity to the minority class, more injury events correctly when compared to traditional learning. Equally important is the fact that cost-sensitive learning also produced the most stable models during the training and testing phases. However, FNN models are an exception to these two findings. In particular, although using cost-sensitive learning on these models provided, in the testing datasets, higher specificity and average accuracy values, sensitively, and GMEAN metrics were lower than traditional learning. By the same token, the differences in the standard deviation between cost-sensitive learning and traditional learning were, despite minimal, sometimes higher.

## Discussion

The current investigation aimed to develop an automatic technique for forecasting injury events. This technique is based on the information from the GPS devices collected throughout games and training sessions combined with other descriptive variables (such as the player’s corridor). In this view, the conducted study allowed us to derive two models. The first model consisted of an AdaBoost classifier, and the second of an SVM. Both models generalized the results with acceptable sensitivity, specificity, and accuracy and were revealed to be stable, thus suggesting their applicability in real-world scenarios.

To the authors’ best knowledge, this is the first study that included the use of the mRMR algorithm to rank the features according to their relevance and redundancy for forecasting injuries. In this view, the AdaBoost required 90 features to detect 78.57% of the actual injury events. On the other hand, the SVM model required only 20 features but obtained a lower sensitivity (71.43%). Nevertheless, the selected variables in both cases enabled the two predictive models with a high explanatory capability for injury events.

The player’s position and type of session (i.e., training or match session) were essential descriptive variables for the two injury prediction models (cf. S1 Table). The player position has not been broadly reported to be associated with an increased injury risk factor [51]; however, injury rates are documented to be higher for matches than training sessions [52]. Possible justifications for the increased injury rate in match sessions may arise from the variations in the intensities of training and competition [53] and, to a certain level, from the type of training before the next match (e.g., tactical practice) [54]. Thus, the two developed models seem to capture this information and use it to predict injuries in conjunction with the other predictors.

The day of the week is another descriptive variable the models require for predicting injuries. To the authors’ best knowledge, no other study on injury prediction in professional football reported a relationship between the day of the week and injury risk. All things considered, the added descriptive variables were essential to leverage the model’s performance in predicting injury and noninjury events.

It is also interesting to note that AdaBoost used the player load and all the velocity and acceleration bands in order to predict injuries. The information about the player load across all axes of accelerometer movement, distance, duration, and effort count performed in all bands makes the model detect when players are in undertraining or overtraining situations. Both situations are boosters of injury events, with some studies reporting a U-shaped curve between these parameters and injury risk [15, 55].

Interestingly, those models do not use the players’ age for forecasting injuries. Indeed, the literature that studied the relationship between age and risk of injury is inconsistent. Some works suggest that the risk of injury increases with age, and others report insufficient evidence to infer a significant effect of age on injury risk [56].

The use of cost-sensitive models is an approach followed by several studies for injury detection (see, e.g., [19, 21]) due to the problem’s imbalanced nature, i.e., due to a significant difference between the number of injury and noninjury events. However, to the authors’ best knowledge, this technique was only employed in studies made based on the data collected on screening battery processes, leaving its use with longitudinal GPS data to be investigated in this work.

In the screening studies [19, 21], cost-sensitive models performed better than traditional learning classifiers. Despite having a different data collection process, this study demonstrates to be also in line with these results; however, AdaBoost coupled with a cost-sensitivity train did not show to be superior to traditional learning in terms of average GMEAN value. A plausible explanation is that, unlike the FNN and SVM models, the AdaBoost algorithm is an accuracy-oriented classification algorithm. Thus, even with a cost-sensitive learning approach and oversampling, the specified cost for the minority class could have been insufficient to incline the boosting strategy to the minority class [57].

The most recent studies investigated, on the other hand, the use of tree-style classifiers for injury detection since these models provide the classification rules and the most critical features for injury prediction (see, e.g., [22, 24, 25]). However, a trade-off between performance and interpretability must be made. As a result, tree-style classifiers have a performance that is usually inferior to other methods. The current work, in its turn, is focused on the performance metrics, leaving the interpretability aspect for future works.

In that view, after conducting the 500 runs for each combination, the AdaBoost and the SVM were the best models identified. These models use cost-sensitive learning and can detect, respectively, 78.57% and 71.43% of the injuries in the testing datasets while keeping an acceptable (>65%) true negative rate. Although there is a known trade-off between correctly predicting more injuries and incorrectly flagging noninjury events [27], the SVM model is the most balanced of the two models, obtaining an Area Under the receiver operating characteristic Curve (AUC) of 0.85.

A comparison between the results reported by the previous state-of-the-art works and the results attained in this work is presented in Table 2. It is important to note that the best model for the studies that reported multiple classifiers was selected based on the highest sensitivity since not all studies reported the metrics used in the current study to compute the GMEAN.

**Table 2:** Comparative analysis between results reported by the state-of-the-art works and the results attained in this work.

Work	Model	Sen. (%)	Spe. (%)	Acc. (%)	AUC (%)
Rossi et al. [22]	Decision tree	80.00	^†^	^†^	0.76
Naglah et al. [23] ^‡^	SVM	82.22	^†^	^†^	0.76
Vallance et al. [24] ^‡^^,^^§^	Gradient Boosting	96.81	^†^	96.75	0.97
Vallance et al. [24] ^‡^^,^^¶^	Gradient Boosting	96.56	^†^	96.63	0.97
Rossi et al. [25] ^§^	Gradient Boosting (XGB)	65.00	^†^	^†^	^†^
**This work**	AdaBoost	78.57	65.02	65.08	0.72
	SVM	71.43	74.19	74.22	0.85

^†^ Information not reported.

^‡^ Data was inferred from the plots.

^^§^^ One-week prediction.

^^¶^^ One-month prediction.

Besides being the first work that combined machine learning with GPS data to predict injuries, the work of Rossi et al. [22] can be considered the most influential in this area. It was established in their work an injury forecaster capable of predicting 80.00% of true injury events. It also provided an interpretable framework between injury risk and training performance. However, the AUC metric suggests that the model might create a significant number of false alarms and thus unnecessarily bench players before the next game or training session. Although this situation is also visible in the proposed AdaBoost model, the SVM model remedies this situation by obtaining a more balanced result between sensitivity and specificity at the expense of lower sensitivity.

The models proposed in this study do not require constant manual data collection to forecast injury events accurately, thus being cost- and time-effective. This, however, is not the case for some studies in the literature that combine GPS data with other pieces of information. For example, Naglah et al. [23] obtained one of the highest sensitivity values. Although meritorious, their proposal uses GPS data combined with players’ questionnaire data. Requiring players to fill out questionnaires frequently is a strategy that can be time-consuming and challenging to incorporate into players’ routines. Furthermore, the increase in sensitivity (about 2%) is not significant to the point of requiring questionnaires. In the same view, Rossi et al. [25] combined GPS data with blood parameters to assess individual psychophysiological responses to training and create an injury forecasting model that predicts injury events in the subsequent seven days. Although only three blood samples were collected, on average, from each player, the post-blood collection procedure is complex, costly, time-consuming, and requires specialized personnel to be conducted. Besides that, unfortunately, they were unable to predict more than 65.00% of the injury events.

Vallance et al. [24] presented the best results in the literature, detecting almost every injury event while balancing sensitivity and specificity. Contrary to the approach presented in the current proposal, Vallance et al. [24] generate injury predictions for the forthcoming week or month. The superior performance of Vallance et al.’s [24] method, when compared to the present approach and other methodologies in the scientific literature, could be attributed to the difference in prediction time frames. Generally, forecasts covering a more extended period tend to be more accurate because they allow for a wider margin of error, leading to less precise predictions. For example, predicting an injury for the next week suggests a potential occurrence at any time during those seven days, which is inherently less precise than a prediction pinpointing a specific day for the potential injury.

The main findings of this study will help the coaching staff to identify football players in high-risk situations for injury and improve their decision-making. This will inevitably leverage the team’s performance, and simultaneously, reduce the club’s economic cost due to injury events.

Besides, it will enable constant monitoring of multiple parameters without manual intervention and the analysis from the coaching staff, which is limited due to the large number of parameters collected by the GPS receivers. Indeed, knowing training and competition effects on the injury risk will also improve the training design and ensure that players receive an adequate training session before and after matches by keeping the correct balance between high and low intensities.

Ultimately, having the possibility to adjust the threshold used to convert continuous injury predictions to discrete labels will enable the coaching staff to draw more informed football tactics. This will enable to control the risk of injury events according to, for example, the team’s position in the championship table.

This study is to be seen in the light of some limitations, which can be, at the same time, possible directions for future works. The number of injuries needed to be larger to test more complex models (eventually, recurrent models such as long short-term memory networks) or to assess the models’ prediction capabilities fully. Additional instances from, e.g., another season or different cohorts (for example, U23 and U19 teams) would remedy this situation. The authors thus highlight that the models were only validated for the analyzed football team.

This study did not specifically measure muscle and body fatigue or include certain types of exercise, such as cardio workouts, that players might have engaged in on their rest days (for example, during the dummy days). These factors might also be connected to the risk of injury and could improve the injury prediction models. Nonetheless, this study aims to develop an automated system to predict injury risks daily throughout a football season without requiring continuous manual data collection, such as that needed for assessing muscle and body fatigue (for example, through self-reports). And, although direct measurements of muscle fatigue or specific activities on off days were not part of the data collected, it is reasonable to assume that the information obtained from the GPS devices provide a reliable indication of the player’s physical condition.

In future studies, it would be beneficial to include additional physiologic parameters of the players (e.g., history of prior injuries and rating of perceived exertion for each session) to enhance the models further. Unfortunately, this information was not available at the time of this study. Moreover, future studies should focus on enhancing model interpretability without significantly sacrificing performance.

## Conclusion

This work used three machine learning methods (SVM, FNN, and AdaBoost) to predict injuries from professional football players. Besides using the information from the GPS receivers, the models incorporated the effect of sudden changes in player load by including dummy days (i.e., records with zeros for all parameters). Descriptive variables, such as player position and day of the week, were also included and showed to leverage the ability of the models for injury prediction.

Before feeding information to the models, features were sorted and selected according to their redundancy and relevance to injury risk using the mRMR method. This procedure revealed the player’s position, type of session, velocity bands, and acceleration bands as essential features for injury prediction.

In turn, the predictive models were shown to be able to accurately detect injuries and noninjuries events, especially the AdaBoost and the SVM, trained with cost-sensitive learning. These models were able to predict more than 70.00% of new injury and noninjury events and be stable in terms of performance metrics. Comparing these results with the ones available in the literature, the models developed in this work stand out for (a) being the most balanced ones (between sensitivity and specificity), (b) not requiring lengthy and manual data collection processes, and (c) the ones that predict injury for short time frames (in this case, one day).

Although the number of injuries was not large enough to fully assess the models’ prediction capabilities, the current models can be used in real-world scenarios. Models will help the coaching staff to identify football players in high-risk situations and, thus, leverage the team’s performance while minimizing rehabilitation costs.

## Supporting information

S1 Table Features’ rank according to the mRMR method. (PDF)

S1 File (ZIP)
