# Supplementary material for: Predicting noncontact injuries of professional football players using machine learning
Source: PLoS One. 2025 Jan 2;20(1):e0315481. doi: 10.1371/journal.pone.0315481 (PMC11694968; doi:10.1371/journal.pone.0315481)
Supplement: S1 Table — (PDF) [file pone.0315481.s001.pdf]

Supplementary Table S1

Features' Rank According to the mRMR Method

GPS and descriptive parameters used in this study, and selected by the low-variance method and sorted according to the mRMR method rank (first column), after using the *k*-means clustering algorithm.

Rank	Feature name
1	Player Corridor 1*
2	Player Position 5*
3	Type of Session*
4	Competition NOS*
5	Velocity Band 3 Recovery Band 5 Total No. Efforts
6	Player Position 2*
7	Day Week 4*
8	Acceleration Band 5 Average Duration
9	Player Position 6*
10	Day Week 6*
11	Velocity Band 4 Max Effort Distance
12	Result 3.0*
13	Day Week 3*
14	Acceleration Band 5 Average Distance
15	Day Week 5*
16	Peak Player Load
17	Acceleration Band 4 Average Duration
18	Player Corridor 2*
19	Player Position 3*
20	Player Load Band 2 Average Effort Count
21	Result 1.0*
22	Result 0.0*
23	Acceleration Band 4 Average Effort Count
24	IMA Impacts Band 6 Count
25	Average Distance
26	Velocity Band 1 Average Duration
27	Acceleration Band 4 Average Distance
28	Player Load Band 1 Average Distance
29	Velocity Band 3 Recovery Band 3 Total No. Efforts
30	Day Week 2*
31	Average Player Load (1D Up)
32	Acceleration Band 5 Average Effort Count
33	IMA Impacts Band 6 Average Count
34	Average Player Load
35	Velocity Band 1 Average Distance
36	Average Player Load (1D Side)
37	RHIE Bout Recovery - Max
38	Velocity Band 4 Average Distance
39	Velocity Band 2 Average Duration
40	Average Player Load (1D Fwd)
41	Velocity Band 4 Average Duration
42	Velocity Band 2 Average Distance
43	Player Load Band 2 Average Duration
44	Average Player Load (Slow)
45	Velocity Band 3 Average Duration
46	Player Load Band 2 Average Distance
47	IMA Impacts Band 3 Average Count
131	Velocity Band 2 Total Duration
132	Player Load Per Minute
133	Velocity Band 2 Total Distance
134	Velocity Band 1 Total Distance
135	Velocity Band 4 Distance Band 4 Total No. Efforts
136	Acceleration Band 5 Distance %
137	Velocity Band 5 Distance Band 3 Total No. Efforts
138	Velocity Band 5 Recovery Band 5 Total No. Efforts
139	Player Load (Slow)
140	Player Load Band 5 Total Distance
141	IMA Impacts Band 5 Count
142	Total Acceleration Load
143	Acceleration Band 4 Total Effort Count
144	Velocity Band 4 Total Effort Count
145	Acceleration Band 4 Duration %
146	Acceleration Band 2 Total Distance
147	Velocity Band 4 Distance Band 3 Total No. Efforts
148	Acceleration Band 1 Total Effort Count
149	Velocity Band 5 Distance Band 2 Total No. Efforts
150	Velocity Band 3 Total Effort Count
151	Player Load Band 4 Distance %
152	Velocity Band 2 Total Effort Count
153	Profile Max Velocity
154	IMA Impacts Band 1 Count
155	Velocity Band 2 Recovery Band 1 Total No. Efforts
156	Player Load Band 4 Average Distance
157	Metabolic Power Band 1 Total Duration
158	Velocity Band 6 Max Effort Distance
159	Acceleration Band 5 Total Effort Count
160	Max Vel (% Max)
161	Player Load Band 3 Distance %
162	IMA Impacts Band 2 Average Count
163	Velocity Band 5 Max Effort Distance
164	Acceleration Band 5 Duration %
165	Acceleration Band 3 Total Effort Count
166	Player Load Band 3 Total Effort Count
167	Player Load Band 2 Distance %
168	Velocity Band 6 Average Effort Count
169	Player Load (1D Fwd %)
170	Player Load Band 5 Average Effort Count
171	Player Load Band 2 Duration %
172	Player Load Band 3 Total Distance
173	Acceleration Band 4 Distance %
174	Velocity Band 3 Recovery Band 1 Total No. Efforts
175	Acceleration Band 1 Average Distance
176	Duration (m)*
177	Player Load Band 5 Total Effort Count

Continued on the next page.

Table 0: Continued from the previous page

Rank	Feature name	Rank	Feature name
48	Velocity Band 4 Recovery Band 5 Total No. Efforts	178	Acceleration Band 3 Total Distance
49	Velocity Band 3 Average Distance	179	Player Load Band 3 Total Duration
50	Velocity Band 2 Average Effort Count	180	Player Load Band 4 Duration %
51	Acceleration Band 2 Average Effort Count	181	Player Load Band 4 Average Effort Count
52	Velocity Band 5 Recovery Band 4 Total No. Efforts	182	Player Load Per Metre
53	Player Load Band 6 Total Player Load	183	Velocity Band 5 Total Duration
54	Velocity Band 3 Distance Band 4 Total No. Efforts	184	Player Load Band 3 Total Player Load
55	IMA Impacts Band 3 Count	185	Player Load (1D Up %)
56	Velocity Band 4 Distance %	186	Velocity Band 6 Distance Band 3 Total No. Efforts
57	Velocity Band 3 Average Effort Count	187	Velocity Band 5 Total Distance
58	Velocity Band 2 Distance Band 4 Total No. Efforts	188	Player Load Band 4 Total Distance
59	IMA Impacts Band 4 Count	189	Acceleration Band 5 Total Duration
60	Velocity Band 4 Average Effort Count	190	Velocity Band 6 Average Distance
61	Velocity Band 3 Recovery Band 4 Total No. Efforts	191	Acceleration Band 3 Total Duration
62	IMA Impacts Band 4 Average Count	192	Player Load Band 4 Total Duration
63	Velocity Band 3 Distance %	193	Velocity Band 3 Distance Band 2 Total No. Efforts
64	Player Load Band 1 Average Duration	194	IMA Impacts Band 2 Count
65	Acceleration Band 3 Average Effort Count	195	MII Distance Interval 2
66	Velocity Band 2 Recovery Band 2 Total No. Efforts	196	Player Load Band 4 Total Player Load
67	Velocity Band 3 Duration %	197	Velocity Band 5 Recovery Band 2 Total No. Efforts
68	Average Duration	198	Effort Velocity/Duration
69	Velocity Band 5 Average Effort Count	199	Effort Acceleration/Duration
70	Velocity Band 3 Total Duration	200	Acceleration Band 1 Total Distance
71	Velocity Band 4 Recovery Band 3 Total No. Efforts	201	Player Load Band 3 Duration %
72	Velocity Band 4 Duration %	202	Velocity Band 6 Total Effort Count
73	Acceleration Band 2 Total Effort Count	203	MII Distance Interval 3
74	Velocity Band 3 Total Distance	204	Total Duration
75	Acceleration Band 2 Average Distance	205	Velocity Band 2 Distance Band 1 Total No. Efforts
76	Acceleration Band 3 Average Distance	206	Velocity Band 5 Duration %
77	Player Load Band 5 Distance %	207	Player Load Band 1 Duration %
78	Acceleration Band 5 Total Distance	208	Velocity Band 5 Recovery Band 3 Total No. Efforts
79	Acceleration Band 6 Average Effort Count	209	Velocity Band 6 Recovery Band 4 Total No. Efforts
80	Player Load Band 6 Total Distance	210	Velocity Band 2 Duration %
81	Velocity Band 4 Recovery Band 2 Total No. Efforts	211	Player Load Band 4 Total Effort Count
82	Player Load Band 2 Total Effort Count	212	MII Player Load Interval 3
83	Acceleration Band 4 Total Distance	213	Velocity Band 3 Distance Band 1 Total No. Efforts
84	Acceleration Band 6 Average Distance	214	RHIE Total Bouts
85	Player Load Band 6 Average Distance	215	Velocity Band 6 Total Distance
86	Exertion Index	216	Velocity Band 1 Duration %
87	Metabolic Power Band 1 Total Distance	217	MII Player Load Interval 2
88	Total Distance	218	Player Load (1D Side %)
89	Exertion Index Per Minute	219	Acceleration Band 6 Total Distance
90	Player Load Band 3 Average Effort Count	220	Acceleration Band 6 Total Duration
91	Velocity Band 3 Max Effort Distance	221	Age*
92	Velocity Band 6 Recovery Band 5 Total No. Efforts	222	Acceleration Band 6 Total Effort Count
93	Acceleration Band 1 Average Effort Count	223	Player Load Band 1 Total Duration
94	Velocity Band 2 Distance Band 2 Total No. Efforts	224	Acceleration Band 8 Total Distance
95	Velocity Band 3 Recovery Band 2 Total No. Efforts	225	Velocity Band 1 Total Duration
96	Player Load (1D Up)	226	Acceleration Band 3 Distance %
97	Maximum Velocity	227	Player Load Band 1 Distance %
98	Player Load (1D Fwd)	228	Acceleration Band 7 Average Distance
99	Player Load Band 2 Total Distance	229	Velocity Band 4 Recovery Band 1 Total No. Efforts

Continued on the next page.

Table 0: Continued from the previous page

Rank	Feature name	Rank	Feature name
100	IMA Impacts Band 5 Average Count	230	Max Acceleration
101	Total Player Load	231	Acceleration Band 3 Duration %
102	Velocity Band 4 Total Duration	232	Velocity Band 5 Distance Band 4 Total No. Efforts
103	Velocity Band 3 Distance Band 3 Total No. Efforts	233	RHIE Efforts Per Bout - Max
104	Velocity Band 2 Recovery Band 3 Total No. Efforts	234	Acceleration Density Index
105	Player Load (2D)	235	Velocity Band 5 Recovery Band 1 Total No. Efforts
106	Meterage Per Minute	236	Acceleration Band 7 Average Effort Count
107	IMA Impacts Band 1 Average Count	237	RHIE Effort Recovery - Max
108	Velocity Band 4 Total Distance	238	Velocity Band 6 Distance Band 2 Total No. Efforts
109	Player Load Band 2 Total Duration	239	Velocity Band 5 Distance %
110	Velocity Band 5 Average Distance	240	Velocity Band 1 Distance %
111	Player Load Band 3 Average Distance	241	Velocity Band 6 Distance %
112	Competition Taça*	242	Velocity Band 4 Distance Band 1 Total No. Efforts
113	Player Load Band 1 Total Distance	243	Velocity Band 2 Distance %
114	Player Load Band 5 Duration %	244	Player Position 4*
115	Acceleration Band 4 Total Duration	245	Velocity Band 6 Recovery Band 1 Total No. Efforts
116	Player Load Band 5 Total Player Load	246	Velocity Band 2 Max Effort Distance
117	Velocity Band 2 Distance Band 3 Total No. Efforts	247	Acceleration Band 8 Average Distance
118	Player Load Band 2 Total Player Load	248	Acceleration Band 6 Duration %
119	RHIE Bout Recovery - Min	249	Acceleration Band 7 Total Distance
120	MII Distance Interval 1	250	Acceleration Band 8 Total Effort Count
121	Player Load (1D Side)	251	Velocity Band 2 Recovery Band 4 Total No. Efforts
122	Player Load Band 5 Average Distance	252	Acceleration Band 7 Distance %
123	Day Week 0*	253	Acceleration Band 6 Distance %
124	MII Player Load Interval 1	254	Acceleration Band 7 Total Effort Count
125	Velocity Band 4 Distance Band 2 Total No. Efforts	255	Velocity Band 2 Recovery Band 5 Total No. Efforts
126	Velocity Band 5 Total Effort Count	256	Velocity Band 6 Recovery Band 3 Total No. Efforts
127	Player Load Band 1 Total Player Load	257	Velocity Band 6 Recovery Band 2 Total No. Efforts
128	Velocity Band 4 Recovery Band 4 Total No. Efforts	258	Acceleration Band 8 Average Effort Count
129	Player Load Band 3 Average Duration	259	No. Exercises*
130	Player Load Band 6 Total Effort Count	260	Home?*

* Descriptive parameters added by the authors.
